# Theragnostic Glycol Chitosan-Conjugated Gold Nanoparticles for Photoacoustic Imaging of Regional Lymph Nodes and Delivering Tumor Antigen to Lymph Nodes

**DOI:** 10.3390/nano11071700

**Published:** 2021-06-28

**Authors:** In-Cheol Sun, SeongHoon Jo, Diego Dumani, Wan Su Yun, Hong Yeol Yoon, Dong-Kwon Lim, Cheol-Hee Ahn, Stanislav Emelianov, Kwangmeyung Kim

**Affiliations:** 1 Center for Theragnosis, Biomedical Research Institute, Korea Institute of Science and Technology, 5, Hwarang-ro, Seongbuk-gu, Seoul 02792, Korea; pfesun@kist.re.kr (I.-C.S.); jsh@kist.re.kr (S.J.); ip9801@kist.re.kr (W.S.Y.); seerou@kist.re.kr (H.Y.Y.); 2 Department of Materials Science and Engineering, Research Institute of Advanced Materials (RIAM), Seoul National University, 1 Gwanak-ro, Gwanak-gu, Seoul 08826, Korea; chahn@snu.ac.kr; 3 School of Electrical Engineering, University of Costa Rica, San Pedro Montes de Oca, San Jose 11501-2060, Costa Rica; diego.dumani@ucr.ac.cr; 4 KU-KIST Graduate School of Converging Science and Technology, Korea University, 145 Anam-ro, Seongbuk-gu, Seoul 02841, Korea; dklim@korea.ac.kr; 5 School of Electrical and Computer Engineering, Georgia Institute of Technology, 777 Atlantic Drive, Atlanta, GA 30332, USA; 6 Wallace H. Coulter Department of Biomedical Engineering, Georgia Institute of Technology and Emory University School of Medicine, 313 Ferst Drive NW, Atlanta, GA 30318, USA

**Keywords:** theragnostic gold nanoparticle, lymph node, photoacoustic imaging, tumor antigen delivery, ovalbumin, glycol chitosan, immunotherapy

## Abstract

Lymph node mapping is important in cancer immunotherapy because the morphology of lymph nodes is one of the crucial evaluation criteria of immune responses. We developed new theragnostic glycol-chitosan-coated gold nanoparticles (GC-AuNPs), which highlighted lymph nodes in ultrasound-guided photoacoustic (US/PA) imaging. Moreover, the ovalbumin epitope was conjugated GC-AuNPs (OVA-GC-AuNPs) for delivering tumor antigen to lymph node resident macrophage. In vitro studies proved the vigorous endocytosis activity of J774A.1 macrophage and consequent strong photoacoustic signals from them. The macrophages also presented a tumor antigen when OVA-GC-AuNPs were used for cellular uptake. After the lingual injection of GC-AuNPs into healthy mice, cervical lymph nodes were visible in a US/PA imaging system with high contrast. Three-dimensional analysis of lymph nodes revealed that the accumulation of GC-AuNPs in the lymph node increased as the post-injection time passed. Histological analysis showed GC-AuNPs or OVA-GC-AuNPs located in subcapsular and medullar sinuses where macrophages are abundant. Our new theragnostic GC-AuNPs present a superior performance in US/PA imaging of lymph nodes without targeting moieties or complex surface modification. Simultaneously, GC-AuNPs were able to deliver tumor antigens to cause macrophages to present the OVA epitope at targeted lymph nodes, which would be valuable for cancer immunotherapy.

## 1. Introduction

The visualization of the lymphatic system is indispensable in the development of cancer immunotherapy. In particular, the noninvasive assessment of regional lymph nodes is important because they play a critical role in metastasis [1] and immune responses [2]. One of the standard methods to evaluate lymph nodes has been biopsy that is often undesirable because of the invasiveness and inaccuracy [3]. Noninvasive clinical imaging modalities may overcome the limitations of biopsy. For example, positron emission tomography (PET) provides information of lymph nodes with high sensitivity and specificity, based on the enhanced glycolytic activity and metabolism of cancer [4]. Other imaging modalities, such as computed tomography (CT), magnetic resonance imaging (MRI) and ultrasound imaging depict the changes in size and morphology of lymph nodes [5,6]. However, each imaging modality has inherent disadvantages. PET requires a radioactive contrast agent; CT employs ionizing irradiation; MRI is expensive and time-consuming; ultrasound imaging has a low contrast.

Ultrasound-guided photoacoustic (US/PA) imaging is a hybrid imaging technique that overcomes the limitations of ultrasound imaging. Photoacoustic imaging visualizes the thermal expansion of tissues near endogenous or exogenous photoacoustic contrast agents after light irradiation. This imaging mode produces a higher contrast, which conventional ultrasound imaging cannot achieve. In addition, the combination of ultrasound and photoacoustic imaging systems is readily established because both imaging modalities share the same transducer and front-end electronics for acoustic wave detection. As a result, US/PA imaging provides biomedical images with a deeper penetration depth, high spatial resolution, and real-time functional information. For these reasons, researchers have applied US/PA imaging to lymph node mapping [7,8]. However, photoacoustic imaging requires exogenous contrast agents, because endogenous ones, such as hemoglobin, melanin, or lipids, often suffer from strong background absorption or a lack of availability for some diseases [9].

Specially designed photoacoustic contrast agents can improve US/PA imaging qualities of lymph nodes. For example, gold nanoparticles [10,11,12,13], copper neodecanoate nanoparticles [14], carbon nanotubes [15], and organic dyes [16,17] have been developed as photoacoustic imaging contrast agents. Among them, gold nanoparticles have unique optical properties applicable to US/PA imaging. Gold nanoparticles absorb light energy and turn it into a rapid thermal expansion of the tissues and photoacoustic signals upon exposure to the pulsed laser. We can control the absorption wavelength in the near-infrared (NIR) region with the plasmon coupling effect, which appears after the aggregation of gold nanoparticles. These unique properties of gold nanoparticles enable the minimum background signals and maximum penetration depth of US/PA imaging of lymph nodes.

In addition to lymph node imaging, tumor antigen delivery is one of the effective methods of cancer immunotherapy. Even though immunotherapy can overcome the side effects of other cancer treatments, its clinical applications often confront challenges, such as low efficiency and adverse effects [18]. Nanoparticle-based delivery systems designed to target the immune system may increase therapeutic efficiency and decrease off-target side effects. Among various immunotherapy strategies, tumor antigen delivery targets antigen-presenting cells that present the antigen to naïve T cells. Then, the activated CD8^+^ T cells cause anti-cancer immune responses. Nanoparticle-based delivery vehicles increase the immunotherapeutic efficiency by transporting plenty of tumor antigens at once and protecting them until they reach the target sites [19,20].

Here, we designed theragnostic glycol-chitosan-coated gold nanoparticles (GC-AuNPs) for a photoacoustic contrast agent and tumor antigen delivery vehicle. Previously, we demonstrated that gold nanoparticles possessed unique properties suitable for a photoacoustic imaging contrast agent of cancer cell detection [21]. Gold nanoparticles enhance photoacoustic signals after aggregation because it causes elevated local temperature upon laser irradiation and decreased thermal diffusivity of gold nanoparticles. We hypothesize that GC-AuNPs also produce strong photoacoustic signals in the lymph nodes after the endocytic activity of macrophages in lymph nodes. It would overcome the low contrast of ultrasound imaging, while the assessment of lymph nodes with high sensitivity and specificity is possible [22]. In addition, we chemically attach ovalbumin (OVA) epitope as a model antigen on GC-AuNPs to evaluate them as a tumor antigen delivery vehicle, because the lymph-node-resident antigen-presenting cells actively participate in cancer immune reactions [23]. For the feasibility in theragnostic applications, we chose cervical lymph nodes, in which macrophages, inducing antigen-specific T cell responses [24], have been a target for the delivery of antigen. We evaluated the photoacoustic imaging and delivering tumor antigen using GC-AuNPs and OVA-GC-AuNPs in animal models.

## 2. Materials and Methods

### 2.1. Materials

All chemicals in the experiment, such as HAuCl_4_·3H_2_O (99.9%, Sigma-Aldrich, St. Louis, MO, USA), glycol chitosan (MW = 205.22, MP Biomedicals, Santa Ana, CA, USA), OVA epitope (Ac-SIINFEKL-C, Peptron, Daejeon, Korea), N-(3-Dimethylaminopropyl)-N-ethylcarbodiimide hydrochloride (EDC, Sigma-Aldrich, St. Louis, MO, USA), and N-hydroxysuccinimide (NHS, 98%, Sigma-Aldrich, St. Louis, MO, USA), were purchased and used without any purification.

### 2.2. GC-AuNP Synthesis and Characterization

GC-AuNPs were synthesized through the reduction of HAuCl_4_ solution with glycol chitosan as previously reported [25]. Briefly, 300 mL of glycol chitosan solution (1 mg/mL, Sigma-Aldrich Corp., St. Louis, MO, USA) was boiled to 70 °C and mixed with HAuCl_4_·3H_2_O solution (1 mM, 100 mL) under stirring for 24 h until the solution turned to red. GC-AuNPs were washed twice through centrifugation (10,000 rpm, 50 min). The nanoparticles were sonicated for less than 1 min for uniform dispersion. As a control, PEGylated AuNPs (PEG-AuNPs) were produced by mixing the same concentration of citrate-reduced AuNP colloid and PEG-SH solution (MW = 5000, 1 mg/mL) for 24 h under stirring, followed by two rounds of centrifuge/washing step. The absorbance spectrum of GC-AuNPs was recorded using a microplate reader (Synergy™ HT, BioTek Instruments, Winooski, VT, USA), operating in the range from 350 nm to 850 nm. Particle size distribution was measured using Zetasizer Nano ZS (Malvern Instruments, Malvern, UK), in which the intensity-weighted diameter of nanoparticles was calculated. The morphology of GC-AuNPs was captured in transmission electron microscope (TEM) images from JEOL 2010-F TEM (JEOL Ltd., Akishima, Tokyo, Japan) operating at 200 kV. TEM samples were prepared on carbon-coated, 200-mesh copper grids by dropping 10 μL of GC-AuNP colloid. Then, the TEM samples were air-dried for 12 h before TEM imaging. Thermal gravimetric analysis was performed at 2050 TGA V5.4A (TA Instruments, New Castle, DE, USA). The carrier gas was nitrogen with a heating rate of 10 °C/min and a maximum temperature of 800 °C. FT-IR spectra were analyzed with iS50 FT-IR Spectrometer (Thermo Fisher Scientific, Waltham, MA, USA).

### 2.3. Ovalbumin Epitope Conjugation to GC-AuNPs (OVA-GC-AuNP)

The C-terminus of the OVA epitope (SIINFEKL) was conjugated with the amine groups of glycol chitosan using EDC/NHS. OVA peptide (481.5 μg), EDC (144 μg), and NHS (86.5 μg) were dissolved in 1 mL of distilled water under stirring for 20 min. Then, OVA peptide solution was mixed with 50 mL of GC-AuNP colloid under stirring at room temperature for 24 h. After the reaction, the colloid was centrifuged at 12,000 rpm for 20 min and the supernatant was replaced with distilled water for the removal of excess OVA epitope. The amount of OVA epitope conjugated on a GC-AuNP was calculated with a BCA protein assay kit.

### 2.4. Cellular Uptake and In Vitro Cytotoxicity Test (GC or OVA)

J774A.1 macrophages were cultured in DMEM media containing fetal bovine serum (10%), penicillin (100 U/mL), and streptomycin (100 mg/mL), and seeded at a density of 2 × 10^4^ cells on gelatin-coated coverslips in 6-well plates. After the incubation of the cells with GC-AuNPs (0.05 mg Au/mL), cells were washed twice with phosphate buffer saline (pH 7.4) and fixed with paraformaldehyde solution (4%). The coverslips, on which the cells endocytosed GC-AuNPs, were mounted on a slide glass. The bright- and dark-field images were obtained in a Leica DMI3000B microscope (Leica Microsystems, Wetzlar, Germany).

The expression of the OVA epitope after cellular uptake was measured with flow cytometry (BD FACSVerse, BD Bioscience, San Jose, CA, USA). Macrophages (10^6^ cells/dish) were cultured with 100 μg Au/mL of OVA-GC-AuNP for 24 h. Then, phycoerythrin(PE)-labeled SIINFEKL-H-2K^b^ antibody (1 μg per 10^6^ cells) was incubated for 1 h in an icebox. Cells were centrifuged at 1200 rpm for 3 min to remove excess antibodies. The result was analyzed using the FlowJo software.

For cytotoxicity, cells were cultured in 96-well plates with a density of 5 × 10^3^ cells/well and treated with different concentrations of GC-AuNP or OVA-GC-AuNP (12.5, 25, 50, 100, and 200 μgAu/mL) for 24 h. Then, each well was washed with PBS buffer and measured cell viability using Cell Counting Kit-8 from Sigma-Aldrich (St. Louis, MO, USA).

### 2.5. Cell Phantom Photoacoustic Imaging

For photoacoustic imaging of tissue-mimicking cell phantoms, the macrophages with GC-AuNPs were detached with trypsin/EDTA, fixed in paraformaldehyde solution, and dispersed in 20 μL of gelatin solution. The gelatin solution was prepared with 0.75 g of silica particles (40 μm, U.S. Silica Co., Mill Creek, OK, USA) and 15 g of gelatin, derived from acid-cured porcine skin (Sigma-Aldrich Corp., St. Louis, MO, USA), dissolved in 250 mL of distilled water. Then, the mixture was slowly heated to 45 °C, placed into a vacuum chamber at 21 kPA for 10 min, and cooled down to 4 °C until gelation. The macrophage-gelatin dispersion was placed on the surface of the gelatin solution. The US/PA images of the cell phantoms were obtained using a Vevo LAZR small-animal US/PA imaging system (VisualSonics, Toronto, Canada) operating with a 20-MHz ultrasound array transducer and pulsed laser tunable within 680–950 nm wavelength.

### 2.6. In Vivo Lymph Node Imaging and Immunohistology

Animal experiments were performed as approved by the Institutional Animal Care and Use Committee at the Georgia Institute of Technology under protocol A16018 (approval dates: 18 April 2016–17 April 2019). Healthy nu/nu mice (5 weeks, female) were anesthetized with a combination of isoflurane (1.5–2.0%) and O_2_ (0.5 L/min), and GC-AuNPs or OVA-GC-AuNPs (2.5 mg Au/mL, 80 μL) were injected through the right side of the tongue. Two-dimensional co-registered US/PA images of the cervical lymph nodes were acquired by using a Vevo LAZR small-animal US/PA imaging system (VisualSonics, Toronto, Canada) with a 40-MHz ultrasound array transducer. For a three-dimensional analysis of the US/PA images, the transducer was scanned mechanically over the 5 mm in 0.08 mm steps to acquire a set of cross-sectional images. These images visualized the cervical lymph nodes in a volume of 5 × 14 × 15 mm. Photoacoustic images were captured in NanoStepper™ mode (VisualSonics, Toronto, Canada) where the excitation wavelength of the pulsed laser was changed from 680 nm to 860 nm in steps of 20 nm. During in vivo experiment, respiration rate, heart rate, and body temperature of mice were monitored with a heated electrocardiogram pad (VisualSonics, Toronto, Canada).

After US/PA imaging, mice were euthanized, and their cervical lymph nodes were excised. For histological analysis, the excised lymph nodes were fixed in 10% formalin, processed, and embedded in paraffin. The embedded tissue samples were sectioned in 5 μm thickness and stained with hematoxylin and eosin. The bright- and dark-field microscopic images of the tissue were obtained on a Leica DMI3000B microscope (Leica Microsystems, Wetzlar, Germany). Furthermore, the excised cervical lymph nodes were embedded in OCT compound (FSC 22 Clear, Frozen Section Compound, Leica Biosystems Richmond, Inc., IL, USA) and sectioned at a thickness of 10 μm, fixed in PBS containing 4% paraformaldehyde. The tissue samples were stained with PE-labeled SIINFEKL-H-2K^b^ antibody at 4 °C for 24 h and 4′,6-diamidino-2-phenylindole (DAPI) for 20 min. The fluorescence images of lymph nodes were obtained using a Leica TCS SP8 laser-scanning confocal microscope (Leica Microsystems GmbH, Wetzlar, Germany) with Alexa 546 (573 nm) and Ar (458, 488, 514 nm) lasers. The fluorescence intensity of confocal microscope images of lymph nodes was analyzed using Image-Pro software (accessed on: 1 April 2019) (*n* = 5; Media Cybernetic, Rockville, MD, USA).

## 3. Results

**Figure 1:**
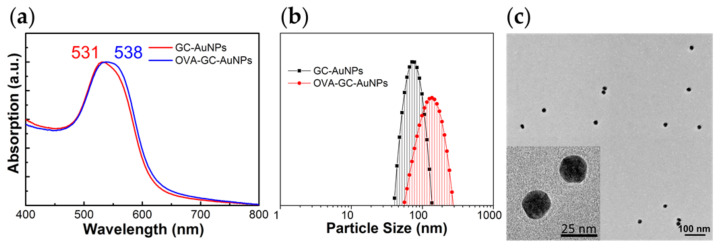
Characterization of GC-AuNPs: (**a**) The UV-vis spectrum presented surface plasmon resonance peak of GC-AuNPs at 531 nm and its red-shift to 538 nm after OVA conjugation to GC-AuNPs (OVA-GC-AuNPs); (**b**) Size distribution of GC-AuNP, measured with DLS, was 94.46 ± 46.45 nm. After OVA conjugation, the size increased to 127.03 ± 23.08 nm; (**c**) The TEM image illustrated the spherical morphology of OVA-GC-AuNPs. The TEM image of GC-AuNPs was indistinguishable from that of OVA-GC-AuNPs (not shown). The discrepancy between TEM images and DLS measurement was due to the hydrophilic property of glycol chitosan and OVA epitope.

We reported the properties of GC-AuNPs in previous studies [21,25,26]. GC-AuNPs exhibited a unique surface plasmon resonance (SPR) peak, positive zeta potential, and spherical morphology. We also investigated the changes in the properties after OVA epitope conjugation. The characterization through UV-vis spectrum, dynamic light scattering (DLS), and zeta potential measurements proved the successful synthesis of OVA-GC-AuNPs. After OVA conjugation, the SPR peak of GC-AuNPs red-shifted from 531 nm to 538 nm in Figure 1a. In addition, we calculated the amount of OVA peptide conjugated to GC-AuNPs with the UV-vis spectrum of the BCA standard curve. According to the calculation, the amount of OVA peptide molecules conjugated to a single nanoparticle was approx. 1400. DLS measurement revealed that the size of GC-AuNPs increased from 94.46 ± 46.45 nm to 127.03 ± 23.08 nm after OVA conjugation in Figure 1b. Zeta potentials of GC-AuNP (4.43 mV) also rose to 26.32 mV (OVA-GC-AuNP). Despite such changes in zeta potentials, nanoparticles dispersed uniformly during the chemical conjugation of OVA peptide. In Figure 1c, TEM images of OVA-GC-AuNPs showed spherical morphology with approx. 20 nm diameter without aggregation. Moreover, the TEM images faintly indicated glycol chitosan coating layer around nanoparticles. In addition, thermal gravimetric analysis showed that the weight ratio of gold to glycol chitosan was 3.82 (27.29%: 7.15%) and FT-IR spectra displayed the disappearance of the peak of the N-H bond after the conjugation of OVA (Figures S1 and S2 in the Supplementary Materials, respectively).

**Figure 2:**
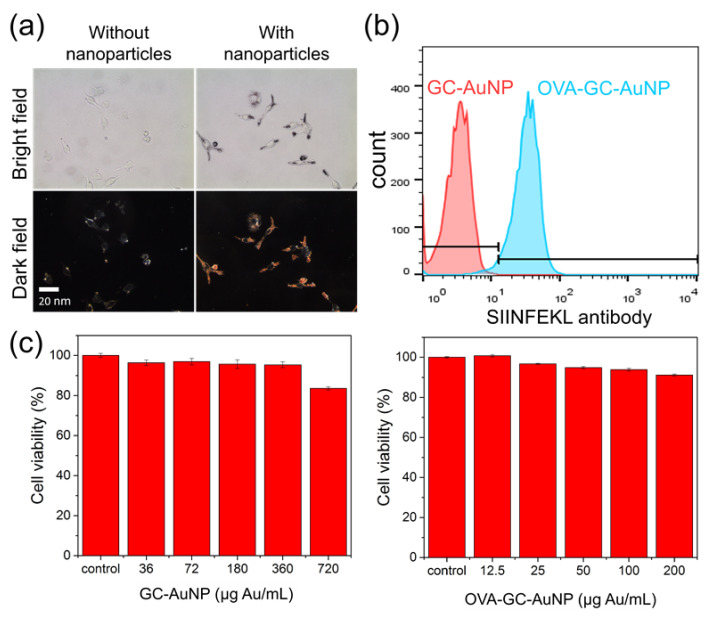
(**a**) Bright- (top) and dark-field (bottom) images of macrophages (J774.A1) after incubation without (left) and with (right) GC-AuNPs. Cells were incubated for 4 h with GC-AuNPs before fixation; (**b**) The expression of OVA epitope on macrophages after the cellular uptake of OVA-GC-AuNP (100 μg Au/mL), measured with flow cytometry; (**c**) Cytotoxicity of GC-AuNPs (left) and OVA-GC-AuNPs (right) according to the concentration.

Next, we tested the cellular uptake of GC-AuNPs in macrophages by incubating them with GC-AuNPs in cell culture media for 4 h. As a control, we prepared samples of macrophages without GC-AuNPs. The dark-field microscopic image of macrophages with GC-AuNPs revealed bright scattering light from nanoparticles in the cytosol area in Figure 2a. Because macrophages actively clear foreign materials [27], many GC-AuNPs were internalized inside macrophages and showed bright signals in dark-field microscopy. In contrast, macrophages without GC-AuNPs did not present any noticeable signals. In addition, bright-field images indicated the biocompatibility of GC-AuNPs because no significant changes in cell morphology or cell death were observed. We also investigated the effect of OVA epitope conjugation on GC-AuNPs. Macrophages exhibited OVA epitope expression after the cellular uptake of OVA-GC-AuNP in Figure 2b. However, it did not aggravate the cellular uptake or increase the cytotoxicity, compared to GC-AuNPs (Figure 2c).

**Figure 3:**
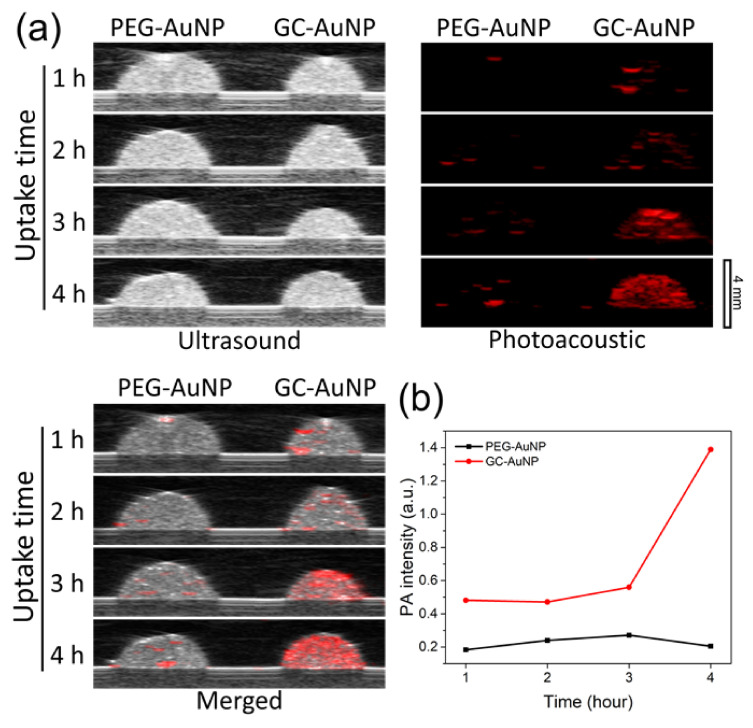
(**a**) Ultrasound (left), photoacoustic (middle), and merged (right) images of macrophage (J774.A1) cell phantoms that were produced after incubation with gold nanoparticles for 1 h, 2 h, 3 h, and 4 h (from the top to the bottom). Each cell phantom contained approx. 13 × 10^5^ cells with the dimension of 4 mm × 4 mm × 2 mm (~15 mm^3^). In PA images, excitation laser wavelength was 680 nm and laser power 0.62 mJ; (**b**) Averaged photoacoustic signal intensity in the cell phantom region according to cellular uptake time.

The US/PA images of the macrophages with GC-AuNPs in tissue-mimicking gelatin phantoms confirmed the feasibility of GC-AuNPs as a photoacoustic contrast agent. In Figure 3a, cell inclusions showed strong photoacoustic signals if macrophages internalized GC-AuNPs for more than 3 h. The intensity of photoacoustic signals increased as cellular uptake time increased. In contrast, cell inclusions of the control group, in which macrophages were incubated with PEGylated AuNPs (PEG-AuNPs), did not exhibit any significant photoacoustic signals, even after 4 h incubation. The graph of averaged photoacoustic signals visualized the effect of cellular uptake time and coating layers on signal intensities from macrophage phantoms (Figure 3b).

**Figure 4:**
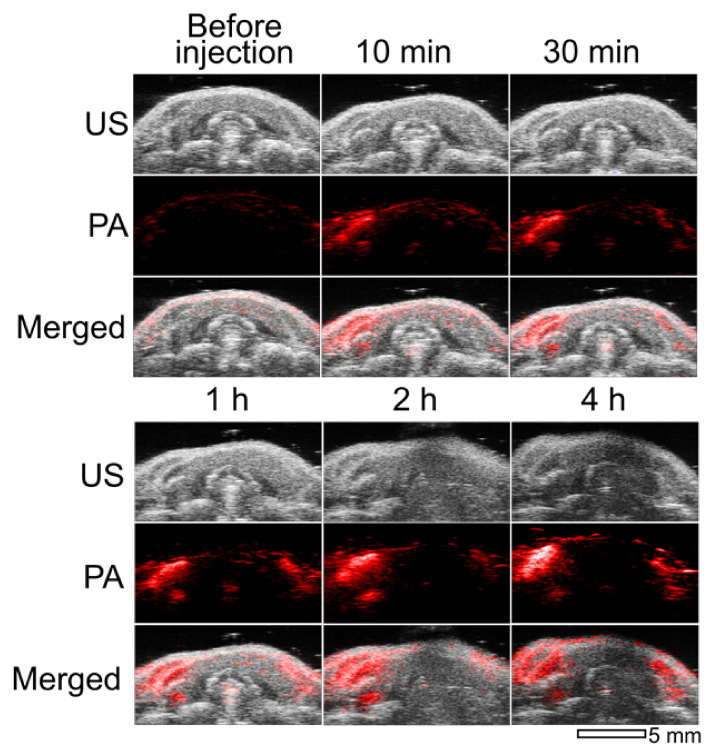
Ultrasound (upper), photoacoustic (middle), and merged images (lower) of cervical lymph nodes of a mouse after injection of GC-AuNPs (2.5 mg Au/mL, 80 μL). Images were obtained before injection, at 10 min, 30 min, 1 h, 2 h, and 4 h of post-injection. Excitation laser wavelength was 680 nm and laser power 0.62 mJ.

We performed in vivo imaging of cervical lymph nodes after the injection of GC-AuNPs into healthy mice on the right side of the tongue. In Figure 4 are US/PA images of cervical lymph nodes according to the post-injection time. Locating the lymph nodes only with ultrasound images was challenging because of their low contrast. On the other hand, photoacoustic images visualized the location of cervical lymph nodes. In addition, these photoacoustic images depicted the accumulation behavior of GC-AuNPs in the lymph nodes. At 10 min after injection, the cervical lymph node on the right side appeared first (the left and right sides of the mouse were reversed in the US/PA images). Then, the left cervical lymph node began to emerge after 1 h (on the right side). The photoacoustic signals from lymph nodes increased up to 4 h post-injection as more GC-AuNPs accumulated in lymph nodes. The increment of signal intensity coincided with previous in vitro results, even though the increase rate was much faster in the case of in vivo imaging than in vitro.

**Figure 5:**
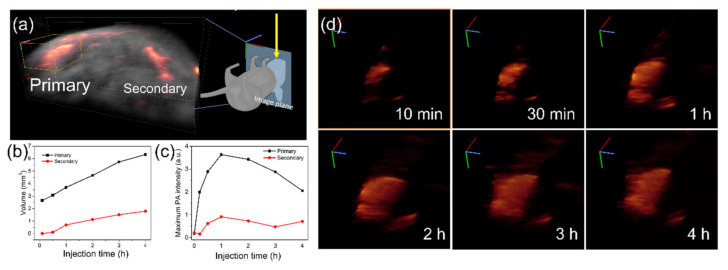
(**a**) Reconstructed three-dimensional images of the lymph nodes from cross-section images; (**b**) Calculated volume of the lymph nodes from reconstructed images; (**c**) Maximum photoacoustic signal intensities from the lymph nodes; (**d**) Detail 3D images of the primary lymph node according to injection time.

We further analyzed the changes of photoacoustic signals with three-dimensional models, which were reconstructed from cross-sectional images. We called the right lymph node, which was closer to the injection site, “primary” and the other one, which was farther from the injection site, “secondary” in Figure 5a. The calculated volumes from both reconstructed lymph node images expanded after the injection (Figure 5b). The graph quantitatively showed that the photoacoustic signals from the primary lymph node were detected at 10 min after injection, then increased up to 4 h. In contrast, signals from the secondary lymph node were delayed until they appeared at 1 h after injection. Moreover, the calculated volume of the secondary lymph node was smaller than that of the primary one. In Figure 5c, the maximum signal intensities in the lymph node images also revealed the behavior of GC-AuNPs in lymph nodes. From both primary and secondary lymph nodes, maximum signal values increased until 1 h after injection, then decreased continuously. It suggested that GC-AuNPs accumulated in a small region of the lymph node with a higher concentration at the early stage. Then, they diffused into a larger area of the lymph node. As seen in the detailed 3D images of Figure 5d, the bright spot in 10 min of post-injection gradually disappeared as the post-injection time passed, while the detected area of the lymph node increased.

**Figure 6:**
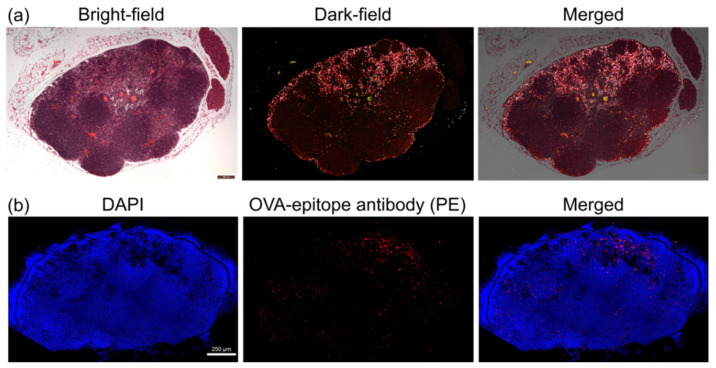
Histological analysis of lymph nodes: (**a**) Bright-field (left), dark-field (middle), and merged (right) microscopic images of a histological sample of the cervical lymph node after the accumulation of GC-AuNPs. In the dark-field images, most signals of GC-AuNPs were found in the subcapsular and medullary sinuses where macrophages are abundant; (**b**) Fluorescent images of the cervical lymph nodes after the injection of OVA-GC-AuNPs. The red fluorescent color indicated the expression of the OVA epitope. Although the OVA epitope was observed throughout the whole lymph node area, the majority centered on medullary sinuses.

After in vivo imaging, we harvested the lymph nodes for histological studies. In the dark-field microscopic images of Figure 6a, most GC-AuNPs accumulated in the subcapsular and medullary sinuses, where macrophages were abundant. The aggregation by cellular uptake in macrophages was the origin of enhanced photoacoustic signals in lymph nodes. On the other hand, fewer GC-AuNPs were present in the B-cell follicles or T-cell zone, where macrophages were relatively scarce. Immunohistostaining analysis revealed the expression of the OVA epitope after the injection of OVA-GC-AuNPs (Figure 6b). After macrophages internalized OVA-GC-AuNPs, they presented tumor antigen (OVA), which appeared as red fluorescence by PE-labeled OVA-epitope antibody. Although the red fluorescence emitted throughout the whole lymph node area, strong signals centered on medullary sinuses where most GC-AuNPs were found.

## 4. Discussion

Typically, gold nanoparticles are synthesized through the reduction of gold(III) chloride using sodium citrate, the most common reducing agent. We produced theranostic gold nanoparticles with glycol chitosan (GC-AuNPs), instead of sodium citrate, because of the electronegative property of glycol chitosan participates in the reduction of gold(III) chloride [28]. Simultaneously, glycol chitosan worked as a coating layer of gold nanoparticles because amine groups of glycol chitosan interacted with the surface of gold nanoparticles. Therefore, the properties of GC-AuNPs were different from those of citrate-reduced gold nanoparticles. For example, zeta-potentials of GC-AuNPs were positive, while that of the citrate-reduced gold nanoparticles were normally negative. In addition, the size discrepancy between TEM images and DLS measurements originated from the hydrophilicity of glycol chitosan on the surface.

The photoacoustic property of gold nanoparticles benefitted from glycol chitosan for the application of lymph node imaging and tumor antigen delivery. The hydrophilicity and biocompatibility of glycol chitosan improved the stability of gold nanoparticles in physiological conditions [26]. This enhanced physiological stability increased the circulation time of GC-AuNPs and extended the photoacoustic imaging time of lymph nodes up to 24 h. In addition, glycol chitosan on the surface of gold nanoparticles endowed positive charges in acidic conditions, due to the pKa value (=6.5) of amine groups [4]. These positive charges of GC-AuNPs may be advantageous for the cellular uptake of macrophages [29]. For the tumor antigen delivery, these amine groups also played an important role in the chemical conjugation of the OVA epitope, while the OVA conjugation maintained the critical properties of GC-AuNPs as a photoacoustic contrast agent.

In vitro studies verified the feasibility of GC-AuNPs as a photoacoustic contrast agent and tumor antigen delivery vehicle. Among various types of immune cells, we chose macrophages because of their vigorous endocytosis activity in lymph nodes [27]. For photoacoustic lymph node imaging and tumor antigen delivery, macrophages should be able to endocytose GC-AuNPs and process the OVA epitope. The dark-field microscopic images illustrated the massive accumulation of GC-AuNPs inside macrophages, due to the active endocytosis of macrophages and the positive charge of GC-AuNPs [29]. We assume that both specific (phagocytosis) and non-specific (macropinocytosis) pathways affected the cellular uptake of GC-AuNPs [30]. The cellular uptake of GC-AuNPs was critical because the enhanced photoacoustic signals relied on the intracellular aggregation, which caused the plasmon coupling effect of gold nanoparticles. As a result of plasmon coupling, the red-shift of SPR peak into the NIR region led to higher absorption efficiency and localized temperature rise [31]. We also utilized the cellular uptake of GC-AuNPs among macrophages as tumor antigen delivery by chemical conjugation of the OVA epitope on GC-AuNPs. The flow cytometry also showed that macrophages presented OVA tumor antigen epitope (SIINFEKL) after cellular uptake and the OVA moieties did not cause any cytotoxicity.

We performed both in vitro and in vivo US/PA imaging using tissue-mimicking cell phantoms with the macrophages and cervical lymph nodes of live mice, respectively. Strong photoacoustic signals were observed from the macrophage cell phantoms if macrophages internalized GC-AuNPs with more than 3 h of incubation time. As mentioned previously, the strong photoacoustic signals originated from the plasmon coupling effect after endocytosis-induced aggregation. In contrast, the cell phantoms with PEG-AuNPs did not present significant photoacoustic signals even after 4 h of cellular uptake, because PEG molecules hindered the nonspecific uptake of gold nanoparticles by macrophages [32]. The cellular uptake of GC-AuNPs by macrophages was the basis of the in vivo lymph node mapping. We obtained in vivo US/PA images of cervical lymph nodes because lymph node resident macrophages endocytosed GC-AuNPs and emitted photoacoustic signals. The combination of two imaging modes generated significant synergy: photoacoustic images differentiated lymph nodes from surrounding tissues with high contrast and low resolution and ultrasound images provided anatomical information of lymph nodes with high resolution and low contrast. Therefore, US/PA imaging and GC-AuNPs were prominent tools for lymph node mapping.

Moreover, the US/PA imaging showed how nanoparticles accumulate in the lymph nodes. Unlike tissue-mimicking cell phantom imaging, intense photoacoustic signals appeared from the lymph node 10 min after injection. We assumed that the early visualization was due to the in vivo environment, in which circulation accelerated the accumulation of GC-AuNPs in lymph nodes. The photoacoustic images displayed another lymph node only 1 h after the injection. The appearance delay of two lymph nodes indicated that drainage of nanoparticles from the injection site was preferential to the lymph node, which was closer to the injection site, and then progressed onto the secondary lymph node. Furthermore, it proved the enhanced stability of GC-AuNPs in the body, because they did not aggregate until they accumulated in the secondary lymph node. Most of all, the dynamic behavior of GC-AuNP in the lymph node was visualized in photoacoustic imaging, which conventional ultrasound imaging would not achieve. The analysis of 3D lymph node images proved that not only did the number of accumulated GC-AuNPs increase according to post-injection time, but also GC-AuNPs diffused throughout the lymph node from the small region with a higher concentration in the early stage of accumulation.

The histological analysis showed the accumulation and spatial distribution of GC-AuNPs or OVA-GC-AuNPs within the lymph node. In the dark-field microscopic images, GC-AuNPs were found in subcapsular and medullar sinuses, where macrophages are abundant. In contrast, few GC-AuNPs existed in B-cell follicles and T-cell zone, where macrophages are scarce. These results confirmed that the origin of photoacoustic signals was GC-AuNPs aggregated in macrophages. Even though GC-AuNPs did not distribute uniformly in the lymph nodes, their photoacoustic signals were strong enough to reveal the morphology of lymph nodes in the photoacoustic images. If OVA-GC-AuNPs were delivered to macrophages, they presented the OVA tumor antigen epitope. The fluorescence of OVA-epitope antibodies was centered on the medullar sinuses, as seen in dark-field microscopy. Therefore, GC-AuNPs demonstrated their feasibility in lymph node mapping and tumor antigen delivery, which would be useful for the application of cancer immunotherapy.

## 5. Conclusions

The feasibility of GC-AuNPs as a photoacoustic contrast agent for lymph node imaging was demonstrated with in vitro and in vivo US/PA imaging. GC-AuNPs presented a superior performance in US/PA imaging of lymph nodes by the active endocytosis of macrophages. GC-AuNPs maintained their stability until they were aggregated inside lymph nodes and resulted in enhanced photoacoustic signals. Moreover, GC-AuNPs were utilized as a tumor antigen delivery vehicle of the OVA epitope, because amine functional groups on the surface of GC-AuNPs were able to accommodate the tumor antigen. Macrophages presented the OVA epitope, which was critical for T cell activation in cancer immunotherapy. This study is substantial in practical terms because we simultaneously achieved lymph node mapping and tumor antigen delivery without using targeting moieties or complex surface modification of nanoparticles. The unique properties of gold nanoparticles, such as high extinction coefficient, high photostability, and large surface-to-volume ratio, and biocompatible and functional properties of glycol chitosan produced excellent properties of GC-AuNPs, suitable for lymph node imaging and tumor antigen delivery for cancer immunotherapy.

## Supplementary Materials

The following are available online at https://www.mdpi.com/article/10.3390/nano11071700/s1, Figure S1: Weight loss profile from thermal gravimetric analysis of GC-AuNPs. Figure S2: FT-IR spectra of before (black line) and after (red line) the conjugation of OVA peptides to GC-AuNPs.
