# Supplementary material for: Theragnostic Glycol Chitosan-Conjugated Gold Nanoparticles for Photoacoustic Imaging of Regional Lymph Nodes and Delivering Tumor Antigen to Lymph Nodes
Source: Nanomaterials (Basel). 2021 Jun 28;11(7):1700. doi: 10.3390/nano11071700 (PMC8307152; doi:10.3390/nano11071700)
Supplement: Supplementary file 1 [file nanomaterials-11-01700-s001.zip › nanomaterials-1270882-supplementary.pdf]

nanomaterials

Supplementary Material

Theragnostic Glycol Chitosan-Conjugated Gold Nanoparticles for Photoacoustic Imaging of Regional Lymph Nodes and Delivering Tumor Antigen to Lymph Nodes

In-Cheol Sun [1,†], SeongHoon Jo [1,2,†], Diego Dumani [3], Wan Su Yun [1,4], Hong Yeol Yoon [1], Dong-Kwon Lim [4], Cheol-Hee Ahn [2], Stanislav Emelianov [5,6,*] and Kwangmeyung Kim [1,4,*]

[1] Center for Theragnosis, Biomedical Research Institute, Korea Institute of Science and Technology, 5, Hwarang-ro, Seongbuk-gu, Seoul 02792, Korea; pfesun@kist.re.kr (I.-C.S.); jsh@kist.re.kr (S.J.); ip9801@kist.re.kr (W.S.Y.) seerou@kist.re.kr (H.Y.Y.)
[2] Department of Materials Science and Engineering, Research Institute of Advanced Materials (RIAM), Seoul National University, 1 Gwanak-ro, Gwanak-gu, Seoul 08826, Korea; chahn@snu.ac.kr
[3] School of Electrical Engineering, University of Costa Rica, San Pedro Montes de Oca, San Jose 11501-2060, Costa Rica; diego.dumani@ucr.ac.cr
[4] KU-KIST Graduate School of Converging Science and Technology, Korea University, 145 Anam-ro, Seongbuk-gu, Seoul 02841, Korea; dklim@korea.ac.kr
[5] School of Electrical and Computer Engineering, Georgia Institute of Technology, 777 Atlantic Drive, Atlanta, GA 30332, USA
[6] Wallace H. Coulter Department of Biomedical Engineering, Georgia Institute of Technology and Emory University School of Medicine, 313 Ferst Drive NW, Atlanta, GA 30318, USA
† These authors contributed equally to this work.
* Correspondence: stas@gatech.edu (S.E.); kim@kist.re.kr (K.K.)

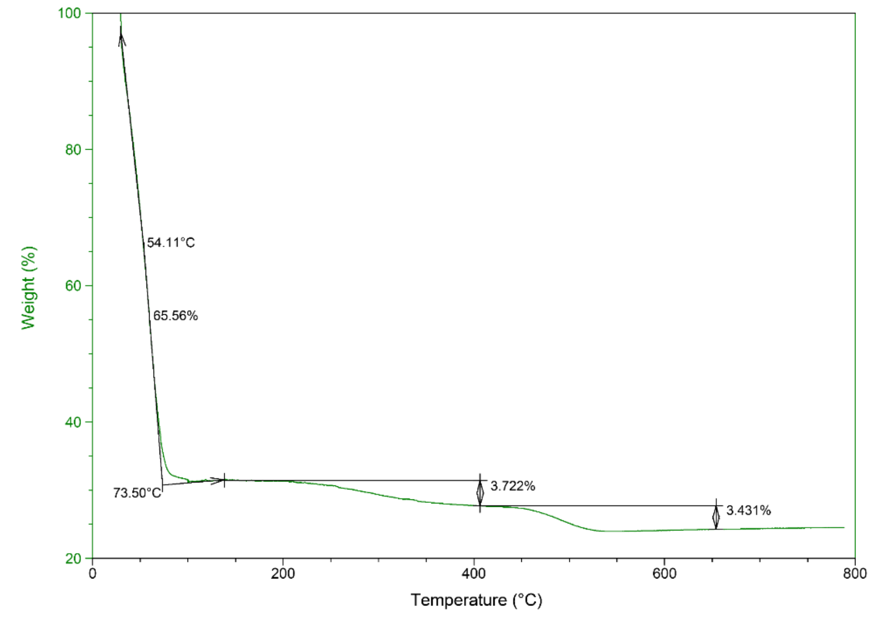

Figure S1. Weight loss profile from thermal gravimetric analysis of GC-AuNPs.

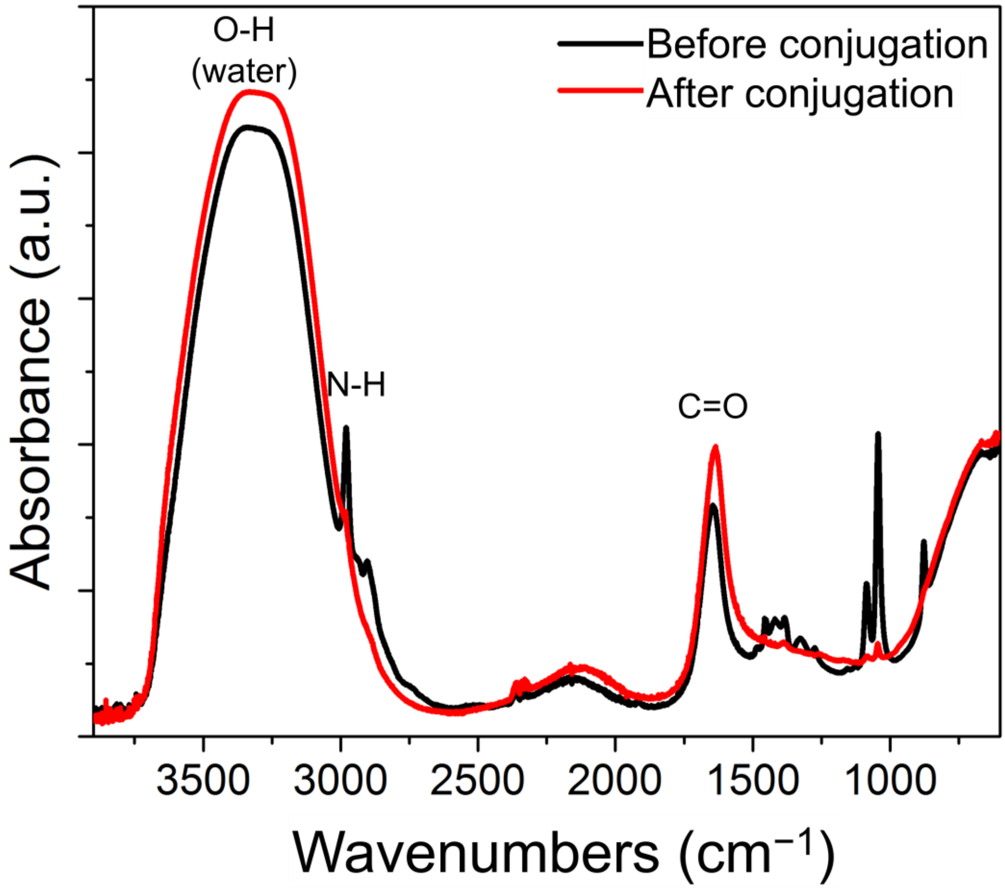

Figure S2. FT-IR spectra of before (black line) and after (red line) the conjugation of OVA peptides to GC-AuNPs.
